# First Reported Case of Reversible Cerebral Vasoconstriction Syndrome After a SARS-CoV-2 Vaccine

**DOI:** 10.7759/cureus.19987

**Published:** 2021-11-29

**Authors:** Josef Finsterer

**Affiliations:** 1 Neurology, Krankenanstalt Rudolfstiftung, Vienna, AUT

**Keywords:** covid-19, stroke, side effect, vaccination, sars-cov-2

## Abstract

This is the first report of reversible cerebral vasoconstriction syndrome (RCVS) as a complication of a SARS-CoV-2 vaccination. A 38-year-old female developed visual impairment due to scotomas and thunderclap headache 18 days after the second shot of the Moderna SARS-CoV-2 vaccine. Multimodal cerebral MRI revealed an acute cortical ischemic lesion in the territory of the right posterior cerebral artery (PCA) on T2-weighted images, diffusion-weighted imaging (DWI), apparent diffusion coefficient (ADC) maps and absence of the PCA on magnetic resonance angiography (MRA). RCVS was diagnosed as the cause of the ischemic lesion. RCVS partially resolved upon nimodipine and anti-seizure drugs within nine days. In conclusion, this case shows that a SARS-CoV-2 vaccination can be followed by RCVS, manifesting as headache, stroke, and epileptiform discharges, and responding favorably to nimodipine.

## Introduction

Anti-SARS-CoV-2 vaccinations have a beneficial effect regarding the mitigation of the SARS-CoV-2 pandemic [1], but can cause side effects in quite a number of patients. Mild-to-severe side effects from SARS-CoV-2 vaccinations occur, irrespective of the product applied and irrespective of the vaccinees' age or gender [2,3]. Adverse reactions to SARS-CoV-2 vaccines are increasingly acknowledged and concern all organs, systems, and tissues [4,5]. The most frequent neurological side effects are headache, myositis, mono- or polyneuritis cranialis, Guillain-Barré syndrome, venous sinus thrombosis, ischemic stroke, hyperactive encephalopathy, Tolosa-Hunt syndrome, and small fiber neuropathy (SFN) [4,5]. Though reversible cerebral vasoconstriction syndrome (RCVS) has been reported as a complication of SARS-CoV-2 infections [6,7,8], it has not been reported as an adverse reaction of a SARS-CoV-2 vaccination. Here we report the first patient with RCVS after an anti-SARS-CoV-2 vaccination.

## Case presentation

**Figure 1:**
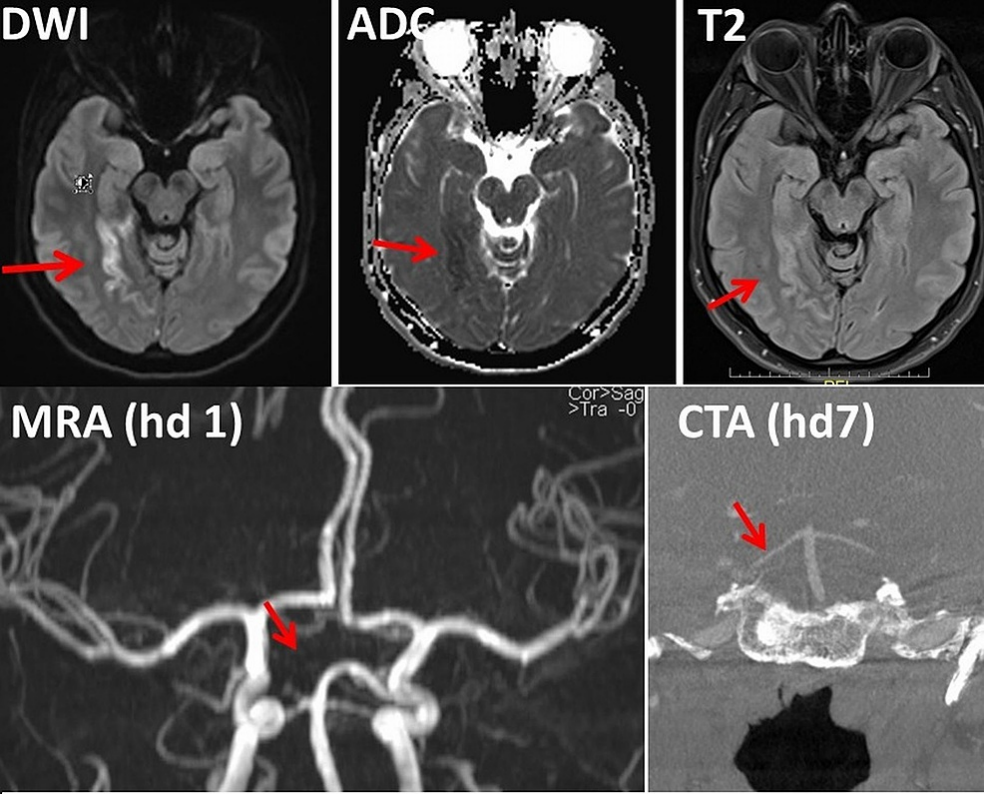
Cerebral MRI and CT angiography of the index patient. MRI showing an ischemic stroke in the territory of the right posterior cerebral artery (upper panels). Magnetic resonance angiography (MRA) on admission shows discontinuation of the right P1 segment (lower left panel). Normal flow in both posterior cerebral arteries (PCAs) was documented on CT angiography (CTA) after seven days of nimodipine, suggesting vasospasm. DWI: Diffusion-weighted imaging, ADC: Apparent diffusion coefficient.

The patient is a 38-year-old female who experienced sudden onset blurred vision bilaterally followed by a focal headache over the right occipital projection one day prior to admission, which responded favorably to analgesics. Blurred vision recurred the next morning with left-sided predominance. She also experienced a sudden onset thunderclap headache after sneezing, which was followed by the same right-sided headache (VAS 3) as one day before. Her previous history was positive for infrequent migraine attacks without aura, smoking (10 P/years), and a second shot of the Moderna SARS-CoV-2 vaccine 18 days prior to symptom onset. A clinical neurological exam on admission revealed blotchy scotoma in the left eye exclusively. Cerebral MRI revealed an acute cortical ischemic lesion in the territory of the right posterior cerebral artery (PCA) (Figure 1). Perimetry disclosed patchy visual field deficits in the left upper quadrant. Work-up for the etiology of the stroke excluded venous sinus thrombosis (VST; normal D-dimer, normal venography), cerebral vasculitis, antiphospholipid antibody syndrome, arterial hypertension, hyperlipidemia, diabetes, and atrial fibrillation. EEG revealed focal slowing and epileptiform discharges over the right occipital projections. RCVS was suspected and the patient was treated with an anti-seizure drug and nimodipine. CT angiography, seven days after admission, revealed a normal-sized right PCA, which was physiologically filled via the basilary artery, confirming the diagnosis RCVS. The patient was discharged nine days after admission with only mild visual impairment with a treatment of levetiracetam (1000 mg/d) and nimodipine (90 mg/d for another five weeks).

## Discussion

The patient is interesting as it is the first report about RCVS following a SARS-CoV-2 vaccination with the Moderna vaccine. Whether there was truly a causal relationship between the vaccination and RCVS, however, remains speculative. Arguments in favor of a causal relation are that no alternative explanation for the RCVS could be pleaded, that RCVS has been repeatedly reported as a complication of SARS-CoV-2 infections [6], that RCVS occurred time-linked to the vaccination, and that RCVS has been listed in one case on the Pfizer-BioNTech vaccine analysis print [7]. RCVS has also been reported after administration of IV immunoglobulins [8]. We do not agree that the case reported by Jumroendararasame C et al. represents a case of RCVS as the patient did not have a headache and as no vasospasm was documented [9]. Arguments against a causal relation are that RCVS has not been previously published in association with an anti-COVID-19 vaccination and that no appropriate pathophysiologic mechanism associated with the vaccination explains RCVS. About half of the RCVS cases occur post-partum or in association with adrenergic or serotoninergic medication [10]. However, it is conceivable that the vaccination increased the sympathetic tone, that the vaccination caused dysautonomia, or that it triggered endothelialitis or focal vasculitis leading to increased vascular tone. Frequent complications of RCVS are ischemic stroke, intracerebral bleeding, subarachnoid bleeding, seizures, or reversible cerebral edema [11]. Two of these features were found in the index patient.

## Conclusions

This case shows that a SARS-CoV-2 vaccination with the Moderna vaccine can be followed by RCVS, manifesting as headache, stroke, and epileptiform discharges, and that RCVS responds favorably to nimodipine.
